# Supplementary material for: Optimizing sowing time and weather conditions for enhanced growth and seed yield of chia (Salvia hispanica L.) in semi-arid regions
Source: PeerJ. 2025 Apr 8;13:e19210. doi: 10.7717/peerj.19210 (PMC11988109; doi:10.7717/peerj.19210)
Supplement: Supplemental Information 1 — Tmax, Maximum temperature; Tmin, Minimum temperature; RH, Relative humidity; BSS, Bright sunshine hours; GDD, Growing degree days. [file peerj-13-19210-s001.docx]

**Table S1**

Prevailing weather parameters during cropping period from sowing to maturity dates (Average values).

| Treatments | Date of sowing | Date of maturity | | Tmax (℃) | | Tmin (℃) | | RH (%) | | BSS (hours/day) | | Total Rainfall (mm) | | Accumulated GDD | | Day length (hr) | |
| --- | --- | --- | --- | --- | --- | --- | --- | --- | --- | --- | --- | --- | --- | --- | --- | --- | --- |
|  |  | 2021-22 | 2022-23 | 2021-22 | 2022-23 | 2021-22 | 2022-23 | 2021-22 | 2022-23 | 2021-22 | 2022-23 | 2021-22 | 2022-23 | 2021-22 | 2022-23 | 2021-22 | 2022-23 |
| S1 | 01-Jul | 19-Nov-21 | 23-Nov-22 | 30.7 | 29.9 | 20.5 | 19.7 | 73.2 | 74.3 | 4.6 | 4.6 | 351.2 | 433.6 | 2211.8 | 2157.6 | 12.1 | 12.1 |
| S2 | 15-Jul | 26-Nov-21 | 29-Nov-22 | 30.5 | 30.0 | 20.3 | 19.4 | 73.0 | 73.2 | 4.9 | 4.9 | 296.8 | 394.0 | 2067.6 | 2027.7 | 12.0 | 12.0 |
| S3 | 01-Aug | 3-Dec-21 | 5-Dec-22 | 30.5 | 30.1 | 20.0 | 18.9 | 72.4 | 71.6 | 5.1 | 5.0 | 338.2 | 353.0 | 1891.0 | 1858.8 | 12.1 | 12.0 |
| S4 | 15-Aug | 6-Dec-21 | 7-Dec-22 | 30.4 | 30.2 | 19.8 | 18.6 | 72.3 | 70.3 | 5.3 | 5.3 | 335.4 | 273.6 | 1730.0 | 1653.1 | 11.8 | 11.9 |
| S5 | 01-Sep | 14-Dec-21 | 17-Dec-22 | 30.4 | 30.1 | 19.3 | 18.0 | 71.4 | 69.3 | 5.4 | 5.3 | 300.6 | 255.8 | 1521.3 | 1527.3 | 11.6 | 11.9 |
| S6 | 15-Sep | 22-Dec-21 | 22-Dec-22 | 30.2 | 30.0 | 18.6 | 17.3 | 70.5 | 66.8 | 5.6 | 5.5 | 241.8 | 186.2 | 1414.0 | 1349.2 | 11.5 | 11.8 |
| S7 | 01-Oct | 3-Jan-22 | 4-Jan-23 | 30.0 | 30.4 | 17.4 | 16.3 | 68.0 | 64.1 | 5.9 | 5.9 | 199.4 | 170.6 | 1301.4 | 1284.2 | 11.2 | 11.2 |
| S8 | 15-Oct | 16-Jan-22 | 16-Jan-23 | 29.5 | 30.3 | 16.4 | 15.1 | 67.4 | 61.8 | 6.0 | 6.0 | 84.8 | 127.6 | 1200.6 | 1190.2 | 11.1 | 11.1 |
| S9 | 01-Nov | 4-Feb-22 | 1-Feb-23 | 28.9 | 30.4 | 15.2 | 14.3 | 67.7 | 60.1 | 6.1 | 6.1 | 84.8 | 1.8 | 1181.2 | 1177.9 | 11.1 | 11.0 |
| S10 | 15-Nov | 18-Feb-22 | 17-Feb-23 | 28.8 | 30.6 | 14.6 | 14.0 | 67.5 | 58.9 | 6.4 | 6.4 | 84.8 | 1.8 | 1123.2 | 1166.5 | 11.1 | 11.1 |
| S11 | 01-Dec | 16-Mar-22 | 15-Mar-23 | 29.9 | 31.5 | 14.3 | 14.0 | 62.2 | 55.2 | 6.9 | 6.9 | 56.4 | 1.8 | 1282.4 | 1344.6 | 11.2 | 11.2 |
| S12 | 15-Dec | 7-Apr-22 | 9-Apr-23 | 31.8 | 32.3 | 15.1 | 14.3 | 57.0 | 53.0 | 7.5 | 7.5 | 0.4 | 11.2 | 1535.8 | 1533.9 | 11.4 | 11.4 |
| S13 | 01-Jan | 3-May-22 | 7-May-23 | 33.9 | 33.3 | 16.9 | 15.7 | 52.7 | 51.0 | 7.9 | 7.9 | 1.4 | 35.2 | 1923.5 | 1859.8 | 11.7 | 11.8 |
| S14 | 15-Jan | 26-May-22 | 28-May-23 | 35.1 | 34.4 | 18.2 | 17.0 | 50.6 | 49.4 | 7.6 | 7.6 | 2.2 | 61.6 | 2188.1 | 2103.5 | 12.0 | 12.0 |
| S15 | 01-Feb | 21-Jun-22 | 25-Jun-23 | 36.0 | 35.1 | 19.7 | 18.5 | 50.9 | 50.1 | 7.3 | 7.3 | 107.4 | 112.2 | 2517.0 | 2414.0 | 12.3 | 12.3 |

Tmax, Maximum temperature; Tmin, Minimum temperature; RH, Relative humidity; BSS, Bright sunshine hours; GDD, Growing degree days.
