# Supplementary material for: Optimizing sowing time and weather conditions for enhanced growth and seed yield of chia (Salvia hispanica L.) in semi-arid regions
Source: PeerJ. 2025 Apr 8;13:e19210. doi: 10.7717/peerj.19210 (PMC11988109; doi:10.7717/peerj.19210)
Supplement: Supplemental Information 2 — *Sowing dates are same in all the treatments for both the years (2021–22 and 2022–23), S1–S15; different sowing dates at 15 days interval [file peerj-13-19210-s002.docx]

**Table S2**

Dates of Sowing and occurrence of chia phenological events during the both the years (2021-22 and 2022-23) of study.

| Sowing | Date of sowing* | Dates of Flower bud appearance | | date of emergence of flower bud | | Date of opening of first flower | | Date of 50% plants flowering | | Date of completion of flowering | | Date of Maturity | | Date of harvest | |
| --- | --- | --- | --- | --- | --- | --- | --- | --- | --- | --- | --- | --- | --- | --- | --- |
|  |  | 2021-22 | 2022-23 | 2021-22 | 2022-23 | 2021-22 | 2022-23 | 2021-22 | 2022-23 | 2021-22 | 2022-23 | 2021-22 | 2022-23 | 2021-22 | 2022-23 |
| S1 | 01-Jul-21 | 15-Sep-21 | 19-Sep-22 | 22-Sep-21 | 2-Oct-22 | 1-Oct-21 | 10-Oct-22 | 8-Oct-21 | 15-Oct-22 | 10-Nov-21 | 14-Nov-22 | 19-Nov-21 | 23-Nov-22 | 25-Nov-21 | 01-Dec-22 |
| S2 | 15-Jul-21 | 23-Sep-21 | 23-Sep-22 | 1-Oct-21 | 2-Oct-22 | 5-Oct-21 | 5-Oct-22 | 16-Oct-21 | 18-Oct-22 | 19-Nov-21 | 18-Nov-22 | 26-Nov-21 | 29-Nov-22 | 30-Nov-21 | 05-Dec-22 |
| S3 | 01-Aug-21 | 1-Oct-21 | 3-Oct-22 | 9-Oct-21 | 9-Oct-22 | 12-Oct-21 | 12-Oct-22 | 24-Oct-21 | 25-Oct-22 | 26-Nov-21 | 26-Nov-22 | 3-Dec-21 | 5-Dec-22 | 09-Dec-21 | 12-Dec-22 |
| S4 | 15-Aug-21 | 7-Oct-21 | 10-Oct-22 | 13-Oct-21 | 13-Oct-22 | 18-Oct-21 | 18-Oct-22 | 26-Oct-21 | 27-Oct-22 | 28-Nov-21 | 30-Nov-22 | 6-Dec-21 | 7-Dec-22 | 13-Dec-21 | 15-Dec-22 |
| S5 | 01-Sep-21 | 13-Oct-21 | 18-Oct-22 | 20-Oct-21 | 22-Oct-22 | 28-Oct-21 | 28-Oct-22 | 13-Nov-21 | 7-Nov-22 | 1-Dec-21 | 14-Dec-22 | 14-Dec-21 | 17-Dec-22 | 19-Dec-21 | 23-Dec-22 |
| S6 | 15-Sep-21 | 23-Oct-21 | 25-Oct-22 | 2-Nov-21 | 2-Nov-22 | 8-Nov-21 | 8-Nov-22 | 17-Nov-21 | 14-Nov-22 | 11-Dec-21 | 16-Dec-22 | 22-Dec-21 | 22-Dec-22 | 29-Dec-21 | 30-Dec-22 |
| S7 | 01-Oct-21 | 5-Nov-21 | 7-Nov-22 | 16-Nov-21 | 17-Nov-22 | 22-Nov-21 | 24-Nov-22 | 3-Dec-21 | 1-Dec-22 | 22-Dec-21 | 24-Dec-22 | 3-Jan-22 | 4-Jan-23 | 08-Jan-22 | 09-Jan-23 |
| S8 | 15-Oct-21 | 20-Nov-21 | 18-Nov-22 | 29-Nov-21 | 29-Nov-22 | 8-Dec-21 | 8-Dec-22 | 17-Dec-21 | 16-Dec-22 | 3-Jan-22 | 5-Jan-23 | 16-Jan-22 | 16-Jan-23 | 23-Jan-22 | 21-Jan-23 |
| S9 | 01-Nov-21 | 10-Dec-21 | 3-Dec-22 | 18-Dec-21 | 22-Dec-22 | 25-Dec-21 | 24-Dec-22 | 2-Jan-22 | 5-Jan-23 | 23-Jan-22 | 22-Jan-23 | 4-Feb-22 | 1-Feb-23 | 11-Feb-22 | 08-Feb-23 |
| S10 | 15-Nov-21 | 26-Dec-21 | 21-Dec-22 | 3-Jan-22 | 3-Jan-23 | 8-Jan-22 | 8-Jan-23 | 17-Jan-22 | 19-Jan-23 | 9-Feb-22 | 8-Feb-23 | 18-Feb-22 | 17-Feb-23 | 25-Feb-22 | 23-Feb-23 |
| S11 | 01-Dec-21 | 11-Jan-22 | 10-Jan-23 | 23-Jan-22 | 23-Jan-23 | 29-Jan-22 | 30-Jan-23 | 25-Feb-22 | 10-Feb-23 | 5-Mar-22 | 6-Mar-23 | 16-Mar-22 | 15-Mar-23 | 21-Mar-22 | 20-Mar-23 |
| S12 | 15-Dec-21 | 29-Jan-22 | 28-Jan-23 | 14-Feb-22 | 14-Feb-23 | 21-Feb-22 | 12-Mar-23 | 2-Mar-22 | 6-Mar-23 | 29-Mar-22 | 28-Mar-23 | 7-Apr-22 | 9-Apr-23 | 11-Apr-22 | 15-Apr-23 |
| S13 | 01-Jan-22 | 19-Feb-22 | 17-Feb-23 | 11-Mar-22 | 1-Mar-23 | 14-Mar-22 | 14-Mar-23 | 27-Mar-22 | 29-Mar-23 | 21-Apr-22 | 24-Apr-23 | 3-May-22 | 7-May-23 | 07-May-22 | 10-May-23 |
| S14 | 15-Jan-22 | 8-Mar-22 | 7-Mar-23 | 28-Mar-22 | 27-Mar-23 | 3-Apr-22 | 2-Apr-23 | 18-Apr-22 | 20-Apr-23 | 13-May-22 | 18-May-23 | 26-May-22 | 28-May-23 | 30-May-22 | 01-Jun-23 |
| S15 | 01-Feb-22 | 30-Mar-22 | 25-Mar-23 | 18-Apr-22 | 18-Apr-23 | 27-Apr-22 | 27-Apr-23 | 12-May-22 | 16-May-23 | 10-Jun-22 | 15-Jun-23 | 21-Jun-22 | 25-Jun-23 | 26-Jun-22 | 30-Jun-23 |

*Sowing dates are same in all the treatments for both the years (2021-22 and 2022-23), S1-S15; different sowing dates at 15 days interval
