# Supplementary material for: Optimizing sowing time and weather conditions for enhanced growth and seed yield of chia (Salvia hispanica L.) in semi-arid regions
Source: PeerJ. 2025 Apr 8;13:e19210. doi: 10.7717/peerj.19210 (PMC11988109; doi:10.7717/peerj.19210)
Supplement: Supplemental Information 4 [file peerj-13-19210-s004.docx]

**Supplementary table 3**: Interaction effect of seed type, date of sowing and year on seed weight per spike, HUE, and Seed yield of chia

| Seed type | Date of sowing* | Seed weight per spike (g) | | | Heat Use efficiency  (kg ha^−1^ ℃ day^−1^) | | | Seed yield  (kg/ha) | | |
| --- | --- | --- | --- | --- | --- | --- | --- | --- | --- | --- |
|  |  | 2021-22 | 2022-23 | Mean  S×D | 2021-22 | 2022-23 | Mean  S×D | 2021-22 | 2022-23 | Mean  S×D |
| W | S1‒1^st^ Jul | 0.527^h-k^ | 0.604^ef^ | 0.565^e^ | 0.28^r^ | 0.32^o-q^ | 0.30^m^ | 617.0^rs^ | 694.2^m-q^ | 655.6^fg^ |
| W | S2‒15^th^ Jul | 0.548^h^ | 0.617^ef^ | 0.582^de^ | 0.33^opq^ | 0.37^mn^ | 0.35^k^ | 688.2^n-q^ | 753.3^g-k^ | 720.7^cd^ |
| W | S3‒1^st^ Aug | 0.56^gh^ | 0.612^ef^ | 0.586^de^ | 0.42^kl^ | 0.42^jk^ | 0.42^i^ | 794.7^d-h^ | 780.4^f-j^ | 787.5^b^ |
| W | S4‒15^th^ Aug | 0.544^hi^ | 0.628^de^ | 0.586^de^ | 0.47^gh^ | 0.45^h-j^ | 0.46^gh^ | 811.6^d-e^ | 746.5^i-j^ | 779.1^b^ |
| W | S5‒1^st^ Sept | 0.544^hi^ | 0.526^h-k^ | 0.535^f^ | 0.53^de^ | 0.45^hijk^ | 0.49^f^ | 812.5^d-f^ | 677.0^o-q^ | 744.8^c^ |
| W | S6‒15^th^ Sept | 0.49^k-n^ | 0.501^j-m^ | 0.495^g^ | 0.58^bc^ | 0.44^i-k^ | 0.51^c-e^ | 814.9^c-f^ | 589.3^st^ | 702.1^de^ |
| W | S7‒1^st^ Oct | 0.497^j-m^ | 0.466^m-p^ | 0.481^gh^ | 0.60^ab^ | 0.43^jk^ | 0.52^cd^ | 793.3^d-h^ | 549.3^tu^ | 671.3^ef^ |
| W | S8‒15^th^ Oct | 0.435^o-q^ | 0.456^n-q^ | 0.445^ij^ | 0.62^a^ | 0.37^mn^ | 0.49^ef^ | 750.9^h-k^ | 439.0^vw^ | 594.9^h^ |
| W | S9‒1^st^ Nov | 0.428^p-r^ | 0.431^p-r^ | 0.429^jk^ | 0.60^ab^ | 0.31^q^ | 0.46^gh^ | 696.1^m-p^ | 366.4^yzA^ | 531.2^i^ |
| W | S10‒15^th^ Nov | 0.42^qr^ | 0.393^rs^ | 0.406^k^ | 0.49^fg^ | 0.26^r^ | 0.38^j^ | 547.9^tu^ | 305.4^BC^ | 426.6^j^ |
| W | S11‒1^st^ Dec | 0.358^st^ | 0.345^tu^ | 0.352^l^ | 0.32^pq^ | 0.23^st^ | 0.27^n^ | 410.4^wx^ | 309.0^BC^ | 359.7^k^ |
| W | S12‒15^th^ Dec | 0.315^uv^ | 0.293^v^ | 0.304^m^ | 0.22^t^ | 0.20^t^ | 0.21^p^ | 338.0^z^ | 311.9^BC^ | 325.0^l^ |
| W | S13‒1^st^ Jan | 0.24^w^ | 0.211^w^ | 0.225^n^ | 0.08^wx^ | 0.12^v^ | 0.10^r^ | 155.8^E^ | 226.2^D^ | 191.0^n^ |
| W | S14‒15^th^ Jan | 0.145^x^ | 0.139^x^ | 0.142^o^ | 0.04^yzA^ | 0.06^xy^ | 0.05^s^ | 91.0^FG^ | 115.9^EF^ | 103.5^o^ |
| W | S15‒1^st^ Feb | 0.136^x^ | 0.138^x^ | 0.137^o^ | 0.02^A^ | 0.02^zA^ | 0.02^t^ | 38.0^h-k^ | 52.1^GH^ | 45.1^p^ |
| B | S1‒1^st^ Jul | 0.711^ab^ | 0.701^b^ | 0.706^ab^ | 0.34^opq^ | 0.34^n-p^ | 0.34^kl^ | 742.4^i-l^ | 740.8^j-l^ | 741.6^c^ |
| B | S2‒15^th^ Jul | 0.727^ab^ | 0.719^ab^ | 0.723^a^ | 0.38^m^ | 0.39^lm^ | 0.39^j^ | 785.9^e-i^ | 795.3^d-h^ | 790.6^b^ |
| B | S3‒1^st^ Aug | 0.742^a^ | 0.705^ab^ | 0.724^a^ | 0.44^ijk^ | 0.45^h-k^ | 0.44^h^ | 835.8^a-d^ | 833.3^a-d^ | 834.5^a^ |
| B | S4‒15^th^ Aug | 0.693^bc^ | 0.740^a^ | 0.717^a^ | 0.50^fg^ | 0.50^e-g^ | 0.50^d-f^ | 858.2^a-c^ | 826.5^b-e^ | 842.4^a^ |
| B | S5‒1^st^ Sept | 0.658^cd^ | 0.720^ab^ | 0.689^b^ | 0.56^c^ | 0.53^de^ | 0.55^ab^ | 874.0^a^ | 808.5^d-f^ | 841.2^a^ |
| B | S6‒15^th^ Sept | 0.629^de^ | 0.692^bc^ | 0.661^c^ | 0.60^ab^ | 0.52^ef^ | 0.56^a^ | 860.7^ab^ | 698.9^l-p^ | 779.8^b^ |
| B | S7‒1^st^ Oct | 0.622^d-f^ | 0.588^fg^ | 0.605^d^ | 0.56^cd^ | 0.52^ef^ | 0.54^b^ | 722.9^k-n^ | 663.0^pq^ | 693.0^de^ |
| B | S8‒15^th^ Oct | 0.533^h-j^ | 0.626^d-f^ | 0.579^de^ | 0.60^ab^ | 0.46^hi^ | 0.53^bc^ | 720.3^k-o^ | 544.1^u^ | 632.2^g^ |
| B | S9‒1^st^ Nov | 0.506^i-l^ | 0.498^j-m^ | 0.502^g^ | 0.63^a^ | 0.39^lm^ | 0.51^c-e^ | 734.2^k-m^ | 461.1^v^ | 597.6^h^ |
| B | S10‒15^th^ Nov | 0.47^l-o^ | 0.463^m-p^ | 0.467^hi^ | 0.58^bc^ | 0.35^no^ | 0.46^g^ | 651.6^qr^ | 405.1^w-y^ | 528.3^i^ |
| B | S11‒1^st^ Dec | 0.422^qr^ | 0.438^o-q^ | 0.43^jk^ | 0.37^mn^ | 0.28^r^ | 0.32^l^ | 481.8^v^ | 374.0^x-z^ | 427.9^j^ |
| B | S12‒15^th^ Dec | 0.37^st^ | 0.438^o-q^ | 0.404^k^ | 0.26^rs^ | 0.21^t^ | 0.23^o^ | 390.2^xy^ | 324.0^A-C^ | 357.1^k^ |
| B | S13‒1^st^ Jan | 0.249^w^ | 0.245^w^ | 0.247^n^ | 0.11^vw^ | 0.15^u^ | 0.13^q^ | 206.3^D^ | 286.5^C^ | 246.4^m^ |
| B | S14‒15^th^ Jan | 0.165^x^ | 0.163^x^ | 0.164^o^ | 0.05^yz^ | 0.05^yz^ | 0.05^s^ | 107.1^F^ | 107.3^F^ | 107.2^o^ |
| B | S15‒1^st^ Feb | 0.161^x^ | 0.149^x^ | 0.155^o^ | 0.02^A^ | 0.02^zA^ | 0.02^t^ | 45.3^H^ | 54.9^GH^ | 50.1^p^ |
