# Supplementary figures and images for: Optimizing sowing time and weather conditions for enhanced growth and seed yield of chia (Salvia hispanica L.) in semi-arid regions
Source: PeerJ. 2025 Apr 8;13:e19210. doi: 10.7717/peerj.19210 (PMC11988109; doi:10.7717/peerj.19210)

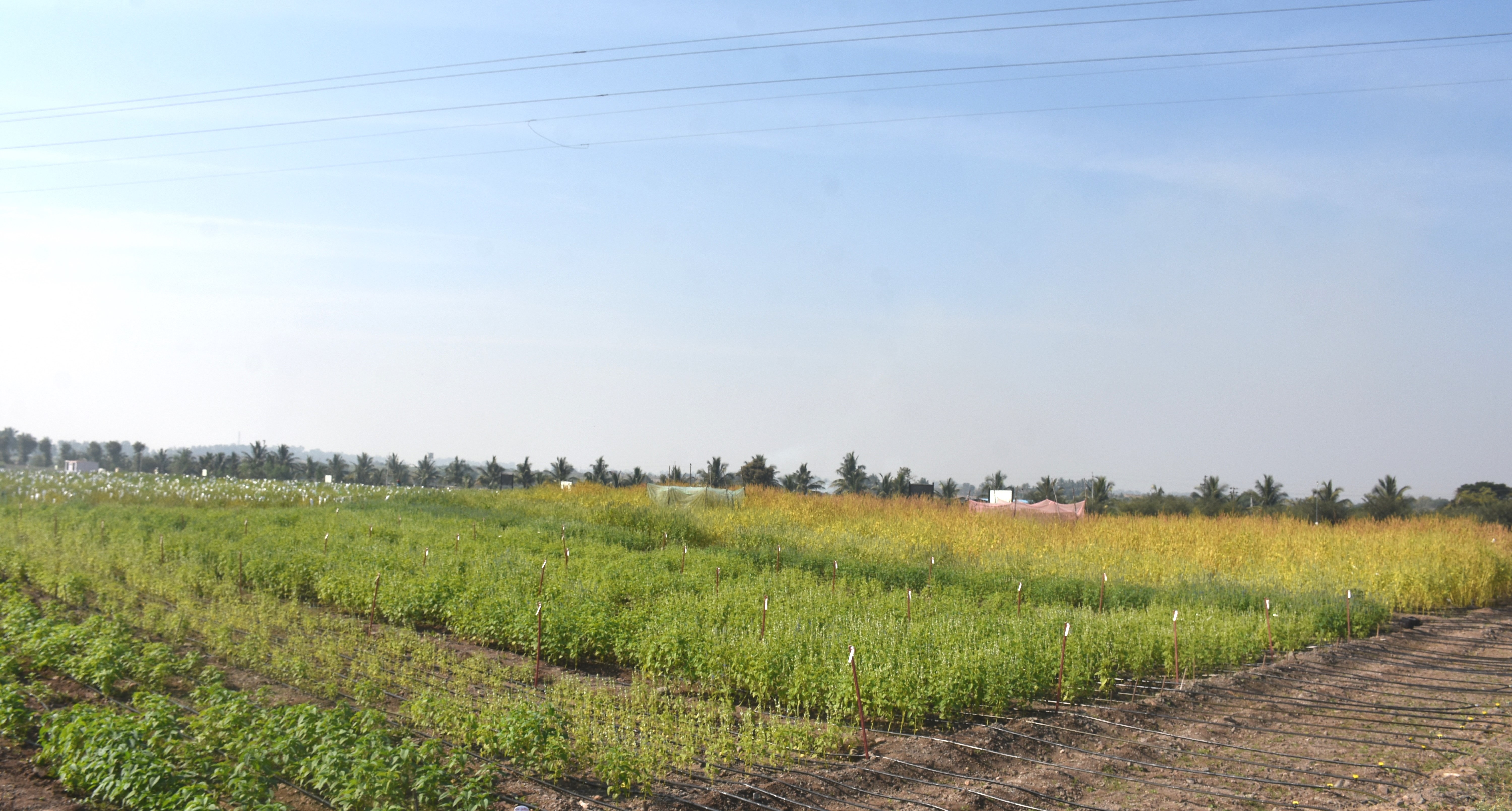

Supplement: Supplemental Information 5 [file peerj-13-19210-s005.jpg]
